# A nomogram for individualized prediction of mild cognitive impairment in patients with subjective cognitive decline during physical examinations: a cross-sectional study

**DOI:** 10.3389/fnagi.2024.1443309

**Published:** 2024-07-03

**Authors:** Tangsheng Zhong, Le Dou, Peiqi Liu, Kexin Huang, Yonghong Wang, Li Chen

**Affiliations:** ^1^School of Nursing, Jilin University, Changchun, China; ^2^First Hospital of Jilin University, Changchun, China

**Keywords:** mild cognitive impairment, subjective cognitive decline, predictive nomogram, cognitive reserve, early detection

## Abstract

**Background and objectives:**

To develop a nomogram for mild cognitive impairment (MCI) in patients with subjective cognitive decline (SCD) undergoing physical examinations in China.

**Methods:**

We enrolled 370 patients undergoing physical examinations at the Medical Center of the First Hospital of Jilin University, Jilin Province, China, from October 2022 to March 2023. Of the participants, 256 were placed in the SCD group, and 74 were placed in the MCI group. The population was randomly divided into a training set and a validation set at a 7:3 ratio. A least absolute shrinkage and selection operator (LASSO) regression model was applied to optimize feature selection for the model. Multivariable logistic regression analysis was applied to construct a predictive model. The performance and clinical utility of the nomogram were determined using Harrell’s concordance index, calibration curves, and decision curve analysis (DCA).

**Results:**

Cognitive reserve (CR), age, and a family history of hypertension were associated with the occurrence of MCI. The predictive nomogram showed satisfactory performance, with a concordance index of 0.755 (95% CI: 0.681–0.830) in internal verification. The Hosmer–Lemeshow test results suggested that the model exhibited good fit (*p* = 0.824). In addition, DCA demonstrated that the predictive nomogram had a good clinical net benefit.

**Discussion:**

We developed a simple nomogram that could help secondary preventive health care workers to identify elderly individuals with SCD at high risk of MCI during physical examinations to enable early intervention.

## 1 Background

With population aging, China’s elderly population will exceed 300 million, accounting for more than 20% of the total population. This represents a stage of moderate population aging; China is expected to enter a stage of severe population aging by approximately 2035 (Mao et al., 2020). Alzheimer’s disease (AD) is the most common type of dementia in the elderly population and is characterized by progressive, irreversible cognitive decline. In China, 15.07 million individuals aged 60 years and over have dementia; it has become the country with the most AD patients in the world, and the number of AD patients is only increasing (Ren et al., 2022). AD is a major threat to the health of China’s elderly population and the sustainable development of China; thus, identification, early diagnosis and treatment of AD are urgently needed. However, drug trials for the prevention and treatment of specific pathophysiological processes of AD have struggled to escape a cycle of failure (Huang et al., 2020). Although the specific causes of failure are extremely complex, a key factor is missing the optimal time for treatment; indeed, irreversible damage to brain tissue has already occurred in patients with mild to moderate AD dementia (Breijyeh and Karaman, 2020). This merits a change in perspective, as prevention may be more important than treatment. Specifically, the focus of dementia research should shift from tertiary prevention (“treat those who are sick”) to secondary prevention (“preventing one case is better than developing ten cures”). Given the current limited understanding of AD, real advances in managing AD may lie in early recognition and intervention (Barnett et al., 2014; Fan and Wang, 2020).

Mild cognitive impairment (MCI) is a prodromal stage of AD, mainly manifesting as varying degrees of mild decline in cognitive functions such as memory, attention, language and visuospatial skills. The annual conversion rate of MCI to AD has reached 10–15%; thus, MCI represents a key stage for the early detection and diagnosis of dementia. More attention to MCI patients may greatly facilitate the secondary prevention of AD. Approximately 12.2% of the elderly population aged 55 years and over in China suffers from MCI, resulting in a high disability rate that is seriously threatening the health of elderly individuals and imposing heavy burdens on family and society. Subjective cognitive decline (SCD), which precedes mild cognitive impairment, is an initial stage in the development of Alzheimer’s disease and is considered the preclinical stage of AD. It is characterized by self-reported decreased memory despite normal cognitive performance. This phenomenon is very common in elderly people. Studies on related markers have also found that the SCD population exhibits similar physiological changes to the AD population, further suggesting that the SCD population is at high risk of AD; SCD has become an international research hotspot for AD, setting off a wave of secondary prevention trials for AD (Fan and Wang, 2020; Pichet Binette et al., 2021). According to a longitudinal follow-up study (Lista et al., 2015), approximately 6.6% of patients with SCD progress to MCI each year, while approximately 2.3% of patients with SCD progress even further to AD. Therefore, in clinical practice, early identification of SCD and MCI populations and effective differentiation of the two during physical examinations are important for secondary prevention of AD. SCD patients misdiagnosed with MCI may receive redundant antidementia therapy, which has small benefits and risks of adverse drug reactions and higher costs. There are also potentially harmful consequences of misdiagnosis with MCI for patients and their families, such as discrimination, stigma and overmedication, leading to increased anxiety or stress and, moreover, cognitive deterioration. In contrast, patients with MCI who are misdiagnosed with SCD are at risk of not receiving appropriate dementia care due to underdiagnosis, delaying treatment of the condition. Therefore, it is highly important to distinguish SCD and MCI in health examinations, determine simple factors for identifying SCD and MCI, and accurately diagnose and administer interventions to MCI patients in a timely manner to reduce the risk of future disease progression.

Millions of people worldwide suffer from MCI, and the diagnosis still relies mainly on highly skilled neurologists, with diagnostic criteria including patients’ medical history, objective cognitive performance, performance on neuropsychological tests such as the Mini-Mental State Examination (MMSE), use of structural MRI to diagnose MCI, or invasive sampling of cerebrospinal fluid. Clinicopathological studies have shown that clinicians have a diagnostic sensitivity ranging from 70.9 to 87.3% and specificity ranging from 44.3 to 70.8%. While MRI data have revealed that MCI produces characteristic brain changes, such as hippocampal and parietal lobe atrophy, these features are thought to lack specificity for MCI. Given this relatively imprecise diagnostic prospect, the invasiveness of CSF sampling and PET scans for diagnosis, and the lack of clinicians specializing in the diagnosis of MCI among secondary prevention personnel in developing countries, simple and convenient diagnostic models are needed that can derive high-precision predictions from practice-wide data. This study provides a new method for determining diagnostic models of SCD and MCI and reports a series of features that can distinguish SCD and MCI based on easily obtained data. These findings are expected to help to screen SCD patients to identify those at high risk of MCI in secondary care settings with few medical personnel and to provide a useful tool for the early diagnosis and treatment of MCI. Finally, the nomogram is used to transform the complex regression equation into a simple and visual graph, so that the results of the prediction model are more readable and have higher use value. A nomogram is a graphical tool that integrates multiple prognostic factors into a single model to predict the probability of a clinical event, such as the development of a disease. It provides an individualized risk assessment based on patient-specific variables, making it a valuable resource for personalized medicine.

## 2 Methods

### 2.1 Study design and participants

This was a cross-sectional study conducted in Northeast China. The China Alzheimer’s Disease Report (2021) identified Jilin Province as the province with the highest incidence of dementia in this region. The study subjects were recruited from the Physical Examination Center of the First Hospital of Jilin University in Northeast China from October 2022 to March 2023. Middle-aged and elderly individuals (aged between 50 and 75 years old) arriving for physical examinations were scheduled to undergo brain computed tomography (CT) or magnetic resonance imaging (MRI) scans. Included individuals had no previous diagnosis of dementia, normal or corrected-to-normal vision and hearing, and were able to communicate normally and participate in the complete neuropsychological tests and imaging examination needed to diagnose SCD and MCI. All subjects provided written informed consent upon entry into the study, and the study was approved by local institutional review boards at all participating sites and registered in the Chinese Clinical Registry (ChiCTR2200055112).

**Figure 1:**
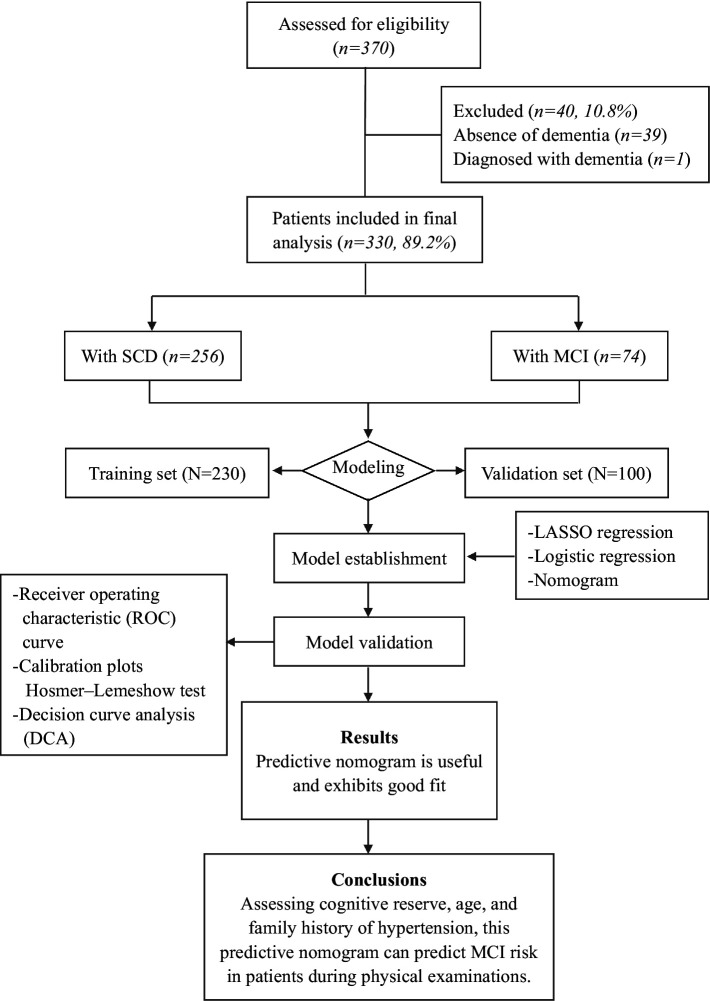
Flowchart of patient enrollment and procedures.

In this study, 370 physical examination subjects were screened, and all subjects were evaluated in terms of neuropsychological criteria. A unified scale was adopted according to the requirements, standardized language was used in the evaluation, and patients with visual, hearing or writing disabilities who could not complete the scale evaluation were excluded (Figure 1). Two neurologists diagnosed those who met the clinical diagnostic criteria with SCD or MCI (Supplementary Table S1).

### 2.2 Data collection

Patients or their close relatives completed a semistructured questionnaire that assessed clinical and cognitive functioning. The interview was conducted by a trained neuropsychological evaluator and lasted approximately 120 min. The sociodemographic information collected included age, sex, ethnicity, education level, occupation, family history, and lifestyle factors.

Variables assessed in the physical examination included muscle strength, thigh circumference, blood pressure, heart rate, grip strength, pace, gait, balance, etc. Patients’ medical history and family history were examined for stroke, diabetes, coronary heart disease, hypertension, hyperlipidemia, cancer, psychiatric disorders, etc.

Neuropsychological tests were used to assess participants’ overall cognitive performance, performance in specific cognitive domains, psychobehavioral symptoms and mental health. These tests included the Mini-Mental State Examination (MMSE), Montreal Cognitive Assessment (MoCA), Activities of Daily Living (ADL) scale, Functional Activities Questionnaire (FAQ), Auditory Verbal Learning Test (AVLT), Shape Alignment Test–Parts A and B (STT-A&B), Memory and Executive Screening (MES), Animal Naming Test (ANT), Clinical Dementia Rating (CDR) scale, Hamilton Depression Scale (HAMD), Hamilton Anxiety Scale (HAMA), and Neuropsychiatric Inventory (NPI). Finally, the results were synthesized to categorize cognitive function according to results on the Clinical Dementia Rating (CDR) scale.

Cognitive reserve (CR): The Cognitive Reserve Scale (CRS) is a new CR assessment that evaluates a person’s participation in cognitively stimulating activities throughout their lifetime (León et al., 2014). The Chinese version of the CRS is a 24-item self-report questionnaire. It is divided into four categories: daily activities, training information, hobbies, and social life. Items are rated on a 5-point Likert scale from 0 (never) to 4 (three or more times per week). It contains three life stages: youth (18–35 years), middle age (36–64 years) and old age (≥65 years). Participants respond to items on the appropriate life-stage subscale for their age. The total score ranges from 0 to 96, with higher scores indicating more frequent participation in activities and higher CR in each life stage. Cognitive reserve scores were calculated at the different life stages (youth, middle age, and old age), and the total CRS score was equal to the score on the stage completed.

### 2.3 Statistical analysis

R 4.0.4 software was used for statistical analysis. The qualitative data are presented as n (%), and the *χ*^2^ test was used for group comparisons. The quantitative data are presented as^−^χ ± s, and the independent-sample *t* test was used for group comparisons. Quantitative data that did not exhibit a normal distribution are described as the median (IQR), and the Mann–Whitney U test was used for group comparisons. The “glmnet” software package was used to establish a LASSO logistic regression model to explore the distinguishing factors of SCD and MCI, and the cross-validation method was used to harmonize the selection of parameter λ. The Bayesian information criterion (BIC) was used to evaluate the goodness-of-fit of the model, and the area under the curve (AUC), Brier score and calibration curve were used to evaluate the discrimination and accuracy of the predictive model. GraphPad Prism software was used to generate a forest plot of the LASSO logistic regression prediction model. The rms package was used to plot the nomogram of the LASSO logistic regression prediction model and the calibration curve of the model. In a two-tailed test, the alpha level was set at 0.05.

## 3 Results

### 3.1 Basic information and single factor analysis

Among the 330 subjects, the mean age was 61.73 ± 7.88 years, 196 were female (59.39%), 319 were married (96.7%), 256 had SCD (77.6%), and 74 had MCI (22.4%). The demographic and clinical characteristics according to group are shown in Tables 1, 2.

**Table 1:** Demographic characteristics of study participants.

Characteristic		All	SCD	MCI	*p* value
		(*n* = 330)	No (*n* = 256)	Yes (*n* = 74)	
Age (years), mean ± SD		61.73 ± 7.88	60.30 ± 7.53	66.68 ± 7.05	<0.001
	<65 years old (%)	218 (66.1)	190 (74.2)	28 (37.8)	<0.001
	≥65 years old (%)	112 (33.9)	66 (25.8)	46 (62.2)	
Ethnicity					0.393
	Han Chinese	311 (94.2)	243 (94.9)	68 (91.9)	
	Other	19 (5.8)	13 (5.1)	6 (8.1)	
Male sex, %		134 (40.6)	97 (37.9)	37 (50.0)	0.080
Education level, %					0.023
	Lower education	85 (25.5)	58 (22.7)	27 (36.5)	
	Higher education	245 (74.2)	198 (77.3)	47 (63.5)	
Occupation, %					0.052
	Physical	70 (21.2)	48 (18.8)	22 (29.7)	
	Intellectual	260 (78.8)	208 (81.3)	52 (70.3)	
Marital status, %					0.019
	Married	319 (96.7)	251 (98.0)	68 (91.9)	
	Other	11 (3.3)	5 (2.0)	6 (8.1)	
History of solitude, %					0.263
	No	298 (90.3)	234 (91.4)	64 (86.5)	
	Yes	32 (9.7)	22 (8.6)	10 (13.5)	
Lifestyle factors					
	Smoking	58 (17.6)	41 (16.0)	17 (23.0)	0.224
	Drinking alcohol	93 (28.2)	66 (25.8)	27 (36.5)	0.079
	Drinking tea/coffee	169 (51.2)	131 (51.2)	38 (51.4)	1.000
Exercise habit, %					0.372
	No	54 (16.4)	39 (15.2)	15 (20.3)	
	Yes	276 (83.6)	217 (84.8)	59 (79.7)	
Social activities, %					
	Low	115 (34.8)	82 (32.0)	33 (44.6)	0.139
	Medium	152 (46.1)	123 (48.0)	29 (39.2)	
	High	63 (19.1)	51 (19.9)	12 (16.2)	

**Table 2:** Clinical characteristics and performance of the participants.

Characteristic		All	SCD	MCI	*p* value
		(*n* = 330)	No (*n* = 256)	Yes (*n* = 74)	
Cognitive reserve (score), mean ± SD		42.39 ± 11.86	43.47 ± 11.26	38.65 ± 13.14	0.002
Step test, %					0.020
	Normal	212 (64.2)	173 (67.6)	39 (52.7)	
	Abnormal	118 (35.8)	83 (32.4)	35 (47.3)	
Five standing tests, %					0.010
	Normal	245 (74.2)	199 (77.7)	46 (62.2)	
	Abnormal	85 (25.8)	57 (22.3)	28 (37.8)	
Grip strength					0.010
	Strong	302 (91.5)	240 (93.8)	62 (83.8)	
	Weak	28 (8.5)	16 (6.3)	12 (16.2)	
Anthropometric measurements, mean ± SD					
	Head circumference (cm)	55.17 ± 2.44	55.23 ± 2.39	54.95 ± 2.61	0.373
	Height (cm)	164.27 ± 7.56	164.03 ± 7.42	165.09 ± 8.01	0.291
	Weight (kg)	66.42 ± 11.48	66.27 ± 11.40	66.93 ± 11.81	0.662
	Waistline (cm)	84.41 ± 11.17	83.81 ± 11.26	86.47 ± 10.66	0.072
	Hipline (cm)	99.67 ± 7.41	99.34 ± 7.44	100.80 ± 7.22	0.136
	Arm circumference (cm)	29.05 ± 3.84	28.95 ± 3.85	29.41 ± 3.82	0.364
	Thigh circumference (cm)	50.00 ± 6.87	49.67 ± 6.44	51.19 ± 8.14	0.094
	Calf circumference (cm)	35.64 ± 3.78	35.67 ± 3.58	35.53 ± 4.42	0.777
Memory loss					0.001
	No	36 (10.9)	28 (10.9)	8 (10.8)	
	Yes	294 (89.1)	228 (89.1)	66 (89.2)	
Decreased concentration					0.125
	No	218 (66.1)	175 (68.4)	43 (58.1)	
	Yes	112 (33.9)	81 (31.6)	31 (41.9)	
Executive dysfunction					0.115
	No	308 (93.3)	242 (94.5)	66 (89.2)	
	Yes	22 (6.7)	14 (5.5)	8 (10.8)	
Lalopathy					0.007
	No	315 (95.5)	249 (97.3)	66 (89.2)	
	Yes	15 (4.5)	7 (2.7)	8 (10.8)	
Disorientation					0.019
	No	316 (95.8)	249 (97.3)	67 (90.5)	
	Yes	14 (4.2)	7 (2.7)	7 (9.5)	
Visual impairment					1.000
	No	253 (76.7)	196 (76.6)	57 (77.0)	
	Yes	77 (23.3)	60 (23.4)	17 (23.0)	
Hearing impairment					0.625
	No	262 (79.4)	205 (80.1)	57 (77.0)	
	Yes	68 (20.6)	51 (19.9)	17 (23.0)	
Dysphagia					1.000
	No	320 (97.0)	248 (96.9)	72 (97.3)	
	Yes	10 (3.0)	8 (3.1)	2 (2.7)	
Gait change					0.001
	No	295 (89.4)	237 (92.6)	58 (78.4)	
	Yes	35 (10.6)	19 (7.4)	16 (21.6)	
Sleep disturbances					0.757
	No	241 (73.0)	188 (73.4)	53 (71.6)	
	Yes	89 (27.0)	68 (26.6)	21 (28.4)	
Present illness					
	Stroke	21 (6.4)	14 (5.5)	7 (9.5)	0.276
	Hypertension	101 (30.6)	74 (28.9)	27 (36.5)	0.252
	Diabetes	52 (15.8)	32 (12.5)	20 (27.0)	0.004
	Dyslipidemia	118 (35.8)	91 (35.5)	27 (36.5)	0.891
	CHD	58 (17.6)	38 (14.8)	20 (27.0)	0.023
Number of comorbidities, %					0.630
	0	132 (40.0)	100 (39.1)	32 (43.2)	
	1	92 (27.9)	74 (28.9)	18 (24.3)	
	2	54 (16.4)	44 (17.2)	10 (13.5)	
	≥3	52 (15.8)	38 (14.8)	14 (18.9)	
Family history, %					
	Dementia	49 (14.8)	38 (14.8)	11 (14.9)	1.000
	Stroke	77 (23.3)	59 (23.0)	18 (24.3)	0.876
	Hypertension	143 (43.3)	122 (47.7)	21 (28.4)	0.003
	Diabetes	88 (26.7)	69 (27.0)	19 (25.7)	0.882
	Dyslipidemia	55 (16.7)	47 (18.4)	8 (10.8)	0.157
	CHD	89 (27.0)	77 (30.1)	12 (16.2)	0.025
	Cancer	64 (19.4)	59 (23.0)	5 (6.8)	0.002

### 3.2 Multifactor analysis and variable selection

**Figure 2:**
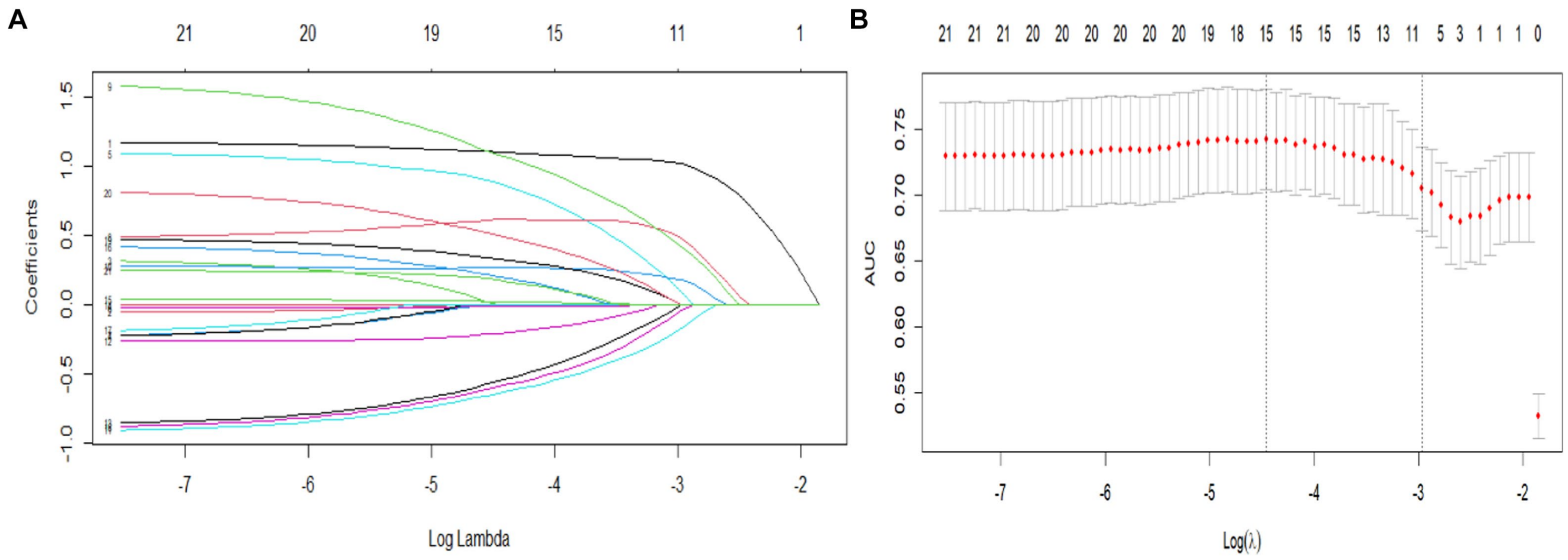
Predictor selection using LASSO regression analysis with 10-fold cross-validation. **(A)** LASSO coefficients from the regression analysis. The tuning parameter (lambda) was used to select the AUC. **(B)** Coefficient profile plot created against the log (lambda) sequence. In the present study, predictor selection was performed according to the 1-SE criteria (right dotted line), and 10 nonzero coefficients were selected.

The data were randomly split into a training set and a validation set in a 7:3 ratio. Factors identified as significant (*p* < 0.10) in the univariate analysis were included as independent variables, and progression to MCI was included as the dependent variable. The LASSO regression and logistic regression models were established using the testing set. The screening of variables according to lambda values from the LASSO logistic regression model is shown in Figure 2. Figure 2A shows the corresponding curve between log(λ) and the number of independent variables; the ordinate shows the mean-square error (MSE) of the model, the lower abscissa shows log(λ), and the upper abscissa shows the number of independent variables in the model with nonzero coefficients corresponding to different log(λ) values. In Figure 2B, the left dashed line represents the MSE minimum optimal harmonic coefficient (λ.min), and the right dashed line represents the MSE within one standard error of the optimal λ (λ.1se). Figure 2B shows the relationship between log(λ) and the LASSO regression coefficient. As λ increased, the degree of compression of the estimated coefficients of each independent variable of the model increased, the coefficient of the independent variable with little impact on the dependent variable was compressed to 0, and the number of independent variables decreased. In this study, λ.1se was selected as the optimal model, and the independent variables included in the logistic regression model were cognitive reserve, age, sex, lalopathy, gait change, thigh circumference, diabetes, alcohol consumption, family history of hypertension, and family history of cancer.

### 3.3 Model construction

**Figure 3:**
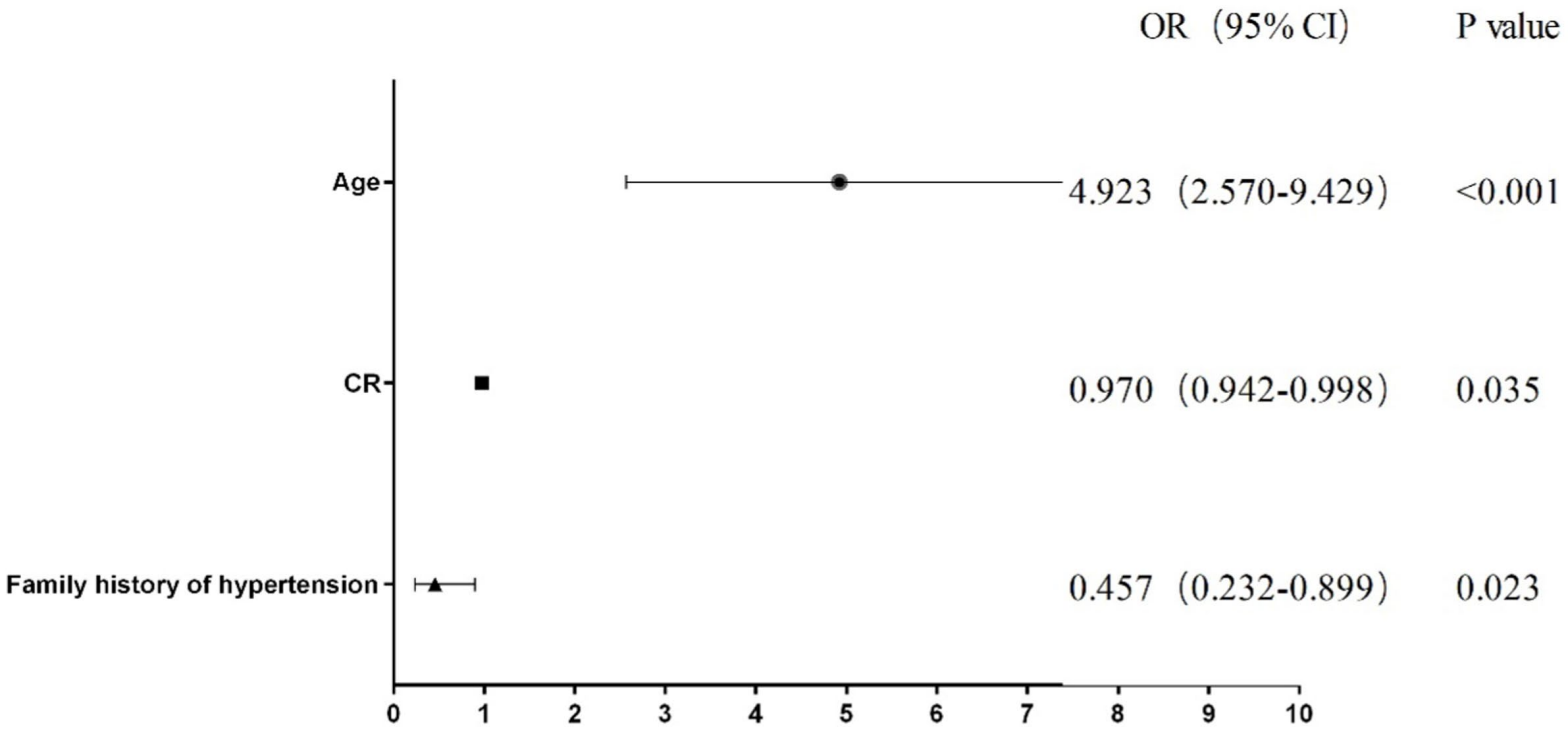
Forest plot of the LASSO logistic regression model.

**Figure 4:**
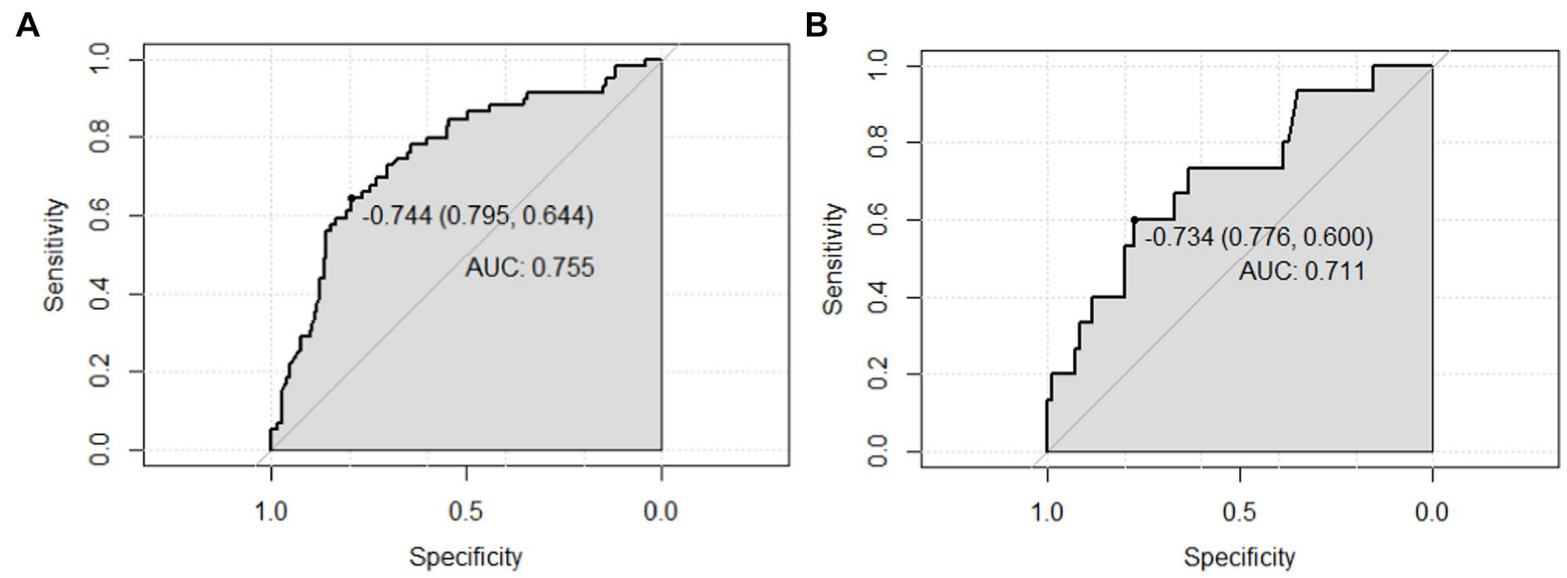
AUC values (representing the discriminatory ability) of the model. **(A)** AUC of the training set, and **(B)** AUC of the validation set. AUC, area under the curve.

**Figure 5:**
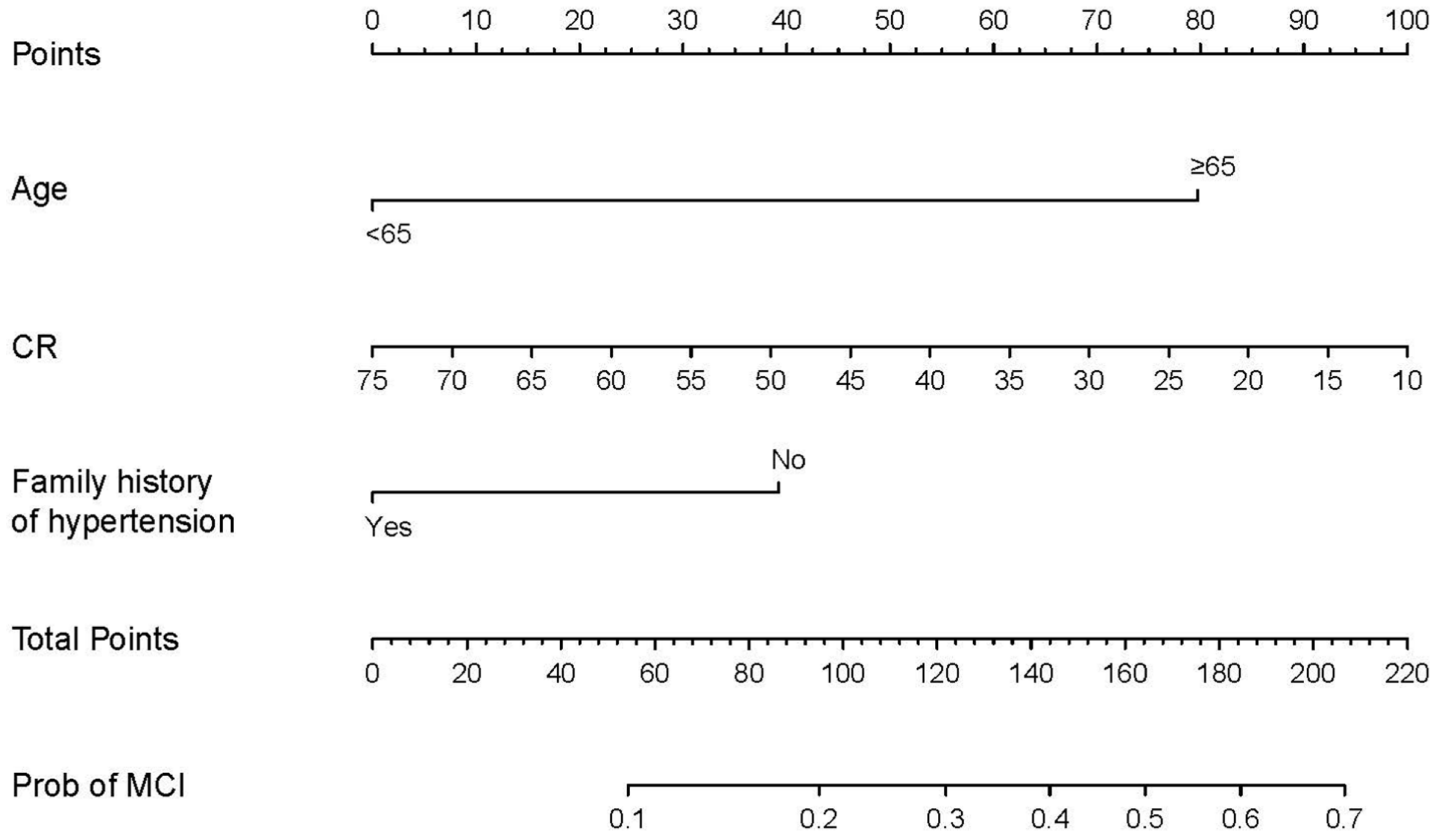
Predictive nomogram for MCI and its algorithm. First, a point was determined for each variable of an SCD patient on the uppermost rule; then, all scores were summed, and the total number of points was calculated. Finally, the corresponding predicted risk of MCI was determined on the lowest rule.

To develop the diagnostic model of MCI, the above 10 variables identified by univariate analysis and LASSO regression were included in the multivariate logistic regression analysis, and the results of the logistic regression model showed that cognitive reserve, age, and family history of hypertension were the factors that distinguished MCI from SCD. The LASSO logistic regression forest plot (Figure 3) visually displays the effects of relevant factors (ORs and 95% CIs). The predictive model had an AUC of 0.755 (95% CI: 0.681–0.830); the internal validation in the validation set yielded a value of 0.711 (95% CI: 0.563–0.859) (Figure 4). A nomogram was constructed to provide a convenient personalized tool for predicting the probability of MCI and visually display the prediction score for MCI (Figure 5). The variables included in the nomogram—age, cognitive reserve (CR) score, and family history of hypertension—were initially identified by univariate analysis and then filtered by LASSO regression and logistic regression analysis, which are considered superior to selecting predictors from univariate analysis. The variables selected for the nomogram were chosen based on their significant associations with mild cognitive impairment (MCI) as identified through univariate and multivariate analyses. Age is a well-known risk factor for cognitive decline. The CR score reflects an individual’s cognitive reserve, which is crucial for buffering against cognitive deterioration. A family history of hypertension has been linked to an risk of cognitive impairment due to its impact on vascular health.

### 3.4 Model evaluation

**Figure 6:**
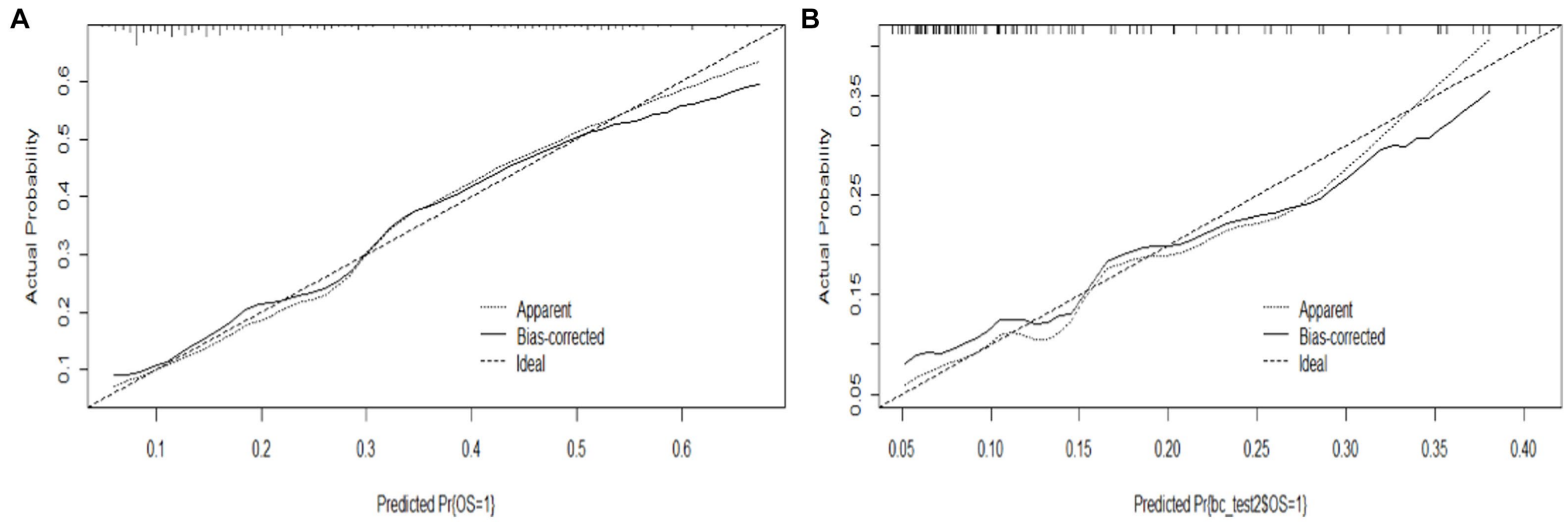
Calibration curve of the model. **(A)** Calibration curve of the training set, and **(B)** calibration curve of the validation set.

The proposed model was well calibrated (Figure 6). The Hosmer–Lemeshow test yielded a nonsignificant *p* value of 0.824 (*p* > 0.05, suggesting that the model exhibited good fit to the data), thereby suggesting that there was no statistical departure from a perfect fit between the predicted and observed values.

**Figure 7:**
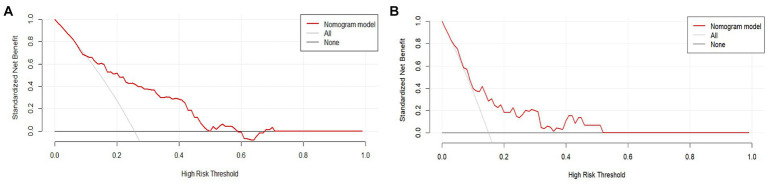
DCA of the nomogram. The solid red line represents the nomogram performance. **(A)** DCA of the training set, and **(B)** DCA of the validation set. DCA, Decision curve analysis.

**Figure 8:**
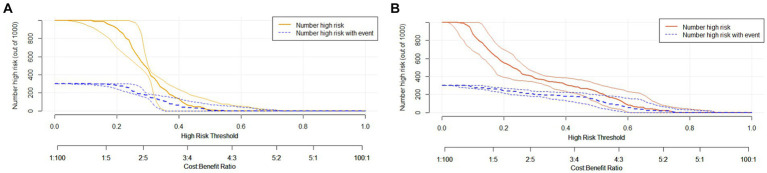
Clinical impact curve of the complex model. Curve interpretation: the yellow **(A)** and red curves **(B)** indicate the number of people classified as positive (high risk) by the simple model **(A)** and the complex model **(B)** at each threshold probability; the blue curve [(number high) risk with outcome] is the number of true positives for each probability threshold.

To assess its clinical usefulness, decision curve analysis (DCA) was also performed. The decision curve based on the nomogram in this study showed that the threshold probability of MCI in SCD patients was 5–50% (Figure 7), and use of this nomogram to predict MCI provided significantly more benefit than either the treat-all scheme or the treat-none scheme. We further used the clinical impact curves to predict the risk stratification of 1,000 people (Figure 8). By comparing the costs and benefits of different treatment options, secondary care doctors select a personalized treatment plan according to the patient’s specific condition.

## 4 Discussion

In the present study, a nomogram was constructed to predict the risk of MCI among SCD patients. This nomogram incorporated 3 variables, namely, age, CR score, and family history of hypertension. The nomogram showed good discriminatory ability, calibration, and clinical usefulness.

Mild cognitive impairment (MCI) is an intermediate state between the cognitive decline associated with healthy aging and dementia, and approximately 12 to 18% of people aged 60 or older are living with MCI (Davis et al., 2018; Xue et al., 2018; Radler et al., 2020). This stage is crucial as approximately one-third of people living with MCI due to Alzheimer’s disease progress to dementia within 5 years. Understanding the prevalence and progression of MCI is essential for developing effective preventive strategies and interventions. Conversely, there is a 53% chance of reversal of MCI to normal cognition after implementing a reasonable intervention (Wood, 2016). Thus, MCI is a high-risk, unstable stage and is the best “intervention window” for AD prevention and treatment. Regardless of country or region, during the physical examination of elderly patients, the possibility of MCI should be routinely considered; when patients complain of memory impairment and other cognitive decline, early identification of MCI and risk prediction is particularly important. However, elderly individuals in China lack health awareness of dementia prevention, and there is a lack of professional health care hospitals and routine primary care examinations in communities. However, the diagnosis of MCI at large medical institutions is very rigorous and requires neuropsychological and imaging data as well as genetic testing. Most of the available prediction models are based on cognitive scale scores and/or neuroimaging data, which are often not feasible to apply in the community or other health care settings and, to some extent, rely on professional use. It is still necessary to develop an effective tool to screen for mild cognitive impairment that also considers individual differences. A potential correlation between cognitive reserve and cognitive performance was found in the early stage of this study, and the various cognitive scores of patients were combined into a quantitative index of cognitive reserve, which addressed such individual differences to a certain extent.

With regard to the risks of MCI, a variety of cognitive habits (Sha et al., 2022), such as personal affairs management (Jiang and Xu, 2014; de Souto Barreto et al., 2018), learning and training (Tarumi et al., 2019; De Wit et al., 2021), and hobbies and social activities (Su et al., 2017; Wang et al., 2020; Zhaoyang et al., 2021), have been explored, but current models for predicting the risk of MCI do not integrate these variables. In addition, several community-based population-based models for predicting MCI have been developed that can improve the diagnosis of MCI (Arevalo-Rodriguez et al., 2021; Lu et al., 2021; McCleery et al., 2021). However, the identification of MCI in these populations is mostly based on the overall cognitive scale score (i.e., classification, not a clinical diagnosis), because most of these classifications only use the overall cognitive scale score, and few use the new standard combined with a separate score on a specific cognitive domain, which – from the perspective of clinical diagnosis – lacks a certain degree of credibility. According to the recent diagnostic criteria for SCD and MCI, it is necessary to conduct separate investigations of various cognitive domains (such as memory, executive function, and language) to assist with clinical diagnosis. In addition, we found few studies that developed models to distinguish between SCD and MCI risks. In the current study, a nomogram for the diagnosis of MCI in SCD patients was constructed based on 3 variables. The variables included in the nomogram were initially identified by univariate analysis and then filtered by LASSO regression and logistics regression analysis, which are considered superior to selecting predictors from univariate analysis (Balachandran et al., 2015). In addition, we assessed the clinical importance of these predictors. With regard to cognitive reserve, a review suggested that the cognitive reserve scale used in the present study is an appropriate tool for assessing the cognitive reserve of the population (Kartschmit et al., 2019), based on a longitudinal assessment considering the frequency of engaging in brain stimulation activities over a lifetime. In terms of sociodemographic characteristics, the cognitive reserve score is not affected by sex, and the possible effect of age is corrected by the computation of the average cognitive reserve score. Therefore, age was controlled for and did not affect cognitive reserve scores, so the results were not biased. In terms of supporting evidence, cognitive reserve refers to the ability of individuals to adaptively use neural networks to compensate for brain damage, which can buffer the negative effect of brain pathology in terms of clinical manifestations, promote the successful response to brain pathology, and ensure the optimization of clinical manifestations or behavioral achievements. The results of this study indicate that the level of cognitive reserve is a protective factor against mild cognitive impairment, and the higher the level of cognitive reserve was, the lower the incidence of mild cognitive impairment.

Regarding age, it has been reported that the incidence of MCI increases with age (Overton et al., 2019; Gaugler et al., 2022). Patients with a family history of hypertension have a low risk of cognitive impairment. Cognitive impairment is often associated with hypertension, but the impact of a family history of hypertension on patients’ cognitive function is modifiable (Qin et al., 2021). This phenomenon may be related to rumination in elderly individuals. Among individuals with a family member with hypertension, the development of a positive perspective is an important determinant of the impact of active rumination on hypertension risk, preventing individuals from suffering from hypertension and cognitive impairment in the future. Family history may be the factor that promotes individuals to actively reflect and re-examine their cognitive function. Such participants may pay more attention to lifestyle factors to reduce the influence of genetic factors on cognitive function, but the cognitive mechanisms underlying the heritability of cognitive dysfunction have yet to be elucidated.

These 3 predictors are easy to obtain clinically, the nomogram had good discriminative ability and calibration, and the DCA results indicate its clinical usefulness. Since cognitive reserve is not yet fully included in public screenings and health awareness, cognitive reserve education is useful for structuring and quantifying patients’ cognitive habits. Therefore, this free nomogram may help community health care institutions and large medical institutions to screen for mild cognitive impairment in patients.

There are some limitations of this study. First, the participants all voluntarily scheduled physical examinations at regional centers in China, and there were regional differences in the prevalence of mild cognitive impairment. Future studies could include external datasets and/or data from multiple centers to verify the results of this study. Second, the selection of predictors was not comprehensive because some indicators are used to diagnose diseases. To avoid diagnostic evaluation bias, this study did not consider biochemical indicators (levels of amyloid, tau protein, etc.), neuroimaging indicators related to MCI, or common MCI screening scales (such as the Montreal Cognitive Assessment). In future studies, more comprehensive factors need to be included to improve prediction accuracy. Second, a classification standard was not identified for the cognitive reserve variable, which would affect the convenience of using the nomogram. Therefore, future research should combine cognitive function and cognitive reserve assessments, which may be more ideal for the identification of MCI, and seek the optimal combination of factors in models to predict MCI risk. Despite these limitations, the study is the first to develop a nomogram based on cognitive reserve to predict the risk of MCI in SCD patients.

## 5 Conclusion

In summary, this study developed a simple nomogram that could help secondary preventive health care workers to identify elderly individuals with SCD at high risk of MCI during physical examinations to enable early intervention. By using this effective tool to screen for MCI in older patients during physical examinations or in the community to promote the early detection of MCI, interventions can be used to prevent MCI and dementia in patients who may have SCD, reducing patient stigma, and optimize medical resource allocation and clinical decision-making. Health management interventions and preventive care for young SCD patients with high cognitive reserves should be improved to inform them of expected future quality of life and reduce the risk of future progression to MCI.

## Data availability statement

The raw data supporting the conclusions of this article will be made available by the authors, without undue reservation.

## Ethics statement

The studies involving humans were approved by the Ethics Committee of School of Nursing, Jilin University. The studies were conducted in accordance with the local legislation and institutional requirements. The participants provided their written informed consent to participate in this study.

## Author contributions

TZ: Conceptualization, Data curation, Formal analysis, Investigation, Software, Writing – original draft, Writing – review & editing. LD: Investigation, Writing – original draft, Writing – review & editing. PL: Writing – original draft, Writing – review & editing, Investigation. KH: Writing – original draft, Writing – review & editing, Investigation. YW: Data curation, Writing – original draft, Writing – review & editing, Resources. LC: Conceptualization, Resources, Supervision, Writing – original draft, Writing – review & editing.

## References

[ref1] Arevalo-Rodriguez I. Smailagic N. Roqué-Figuls M. Ciapponi A. Sanchez-Perez E. Giannakou A.. (2021). Mini-mental state examination (MMSE) for the early detection of dementia in people with mild cognitive impairment (MCI). Cochrane Database Syst. Rev. 2021:CD010783. doi: 10.1002/14651858.CD010783.pub3, PMID: 34313331 PMC8406467

[ref2] Balachandran V. P. Gonen M. Smith J. J. DeMatteo R. P. (2015). Nomograms in oncology: more than meets the eye. Lancet Oncol. 16, e173–e180. doi: 10.1016/S1470-2045(14)71116-7, PMID: 25846097 PMC4465353

[ref3] Barnett J. H. Lewis L. Blackwell A. D. Taylor M. (2014). Early intervention in Alzheimer's disease: a health economic study of the effects of diagnostic timing. BMC Neurol. 14:101. doi: 10.1186/1471-2377-14-101, PMID: 24885474 PMC4032565

[ref4] Breijyeh Z. Karaman R. (2020). Comprehensive review on Alzheimer's disease: causes and treatment. Molecules 25:5789. doi: 10.3390/molecules25245789, PMID: 33302541 PMC7764106

[ref5] Davis M. O'Connell T. Johnson S. Cline S. Merikle E. Martenyi F.. (2018). Estimating Alzheimer's disease progression rates from Normal cognition through mild cognitive impairment and stages of dementia. Curr. Alzheimer Res. 15, 777–788. doi: 10.2174/1567205015666180119092427, PMID: 29357799 PMC6156780

[ref6] de Souto Barreto P. Andrieu S. Rolland Y. Vellas B. DSA MAPT Study Group (2018). Physical activity domains and cognitive function over three years in older adults with subjective memory complaints: secondary analysis from the MAPT trial. J. Sci. Med. Sport 21, 52–57. doi: 10.1016/j.jsams.2017.07.019, PMID: 28802628

[ref7] De Wit L. Chandler M. Amofa P. DeFeis B. Mejia A. O'Shea D.. (2021). Memory support system training in mild cognitive impairment: predictors of learning and adherence. Neuropsychol. Rehabil. 31, 92–104. doi: 10.1080/09602011.2019.1667833, PMID: 31538854 PMC8180362

[ref8] Fan D. Y. Wang Y. J. (2020). Early intervention in Alzheimer's disease: how early is early enough? Neurosci. Bull. 36, 195–197. doi: 10.1007/s12264-019-00429-x, PMID: 31494835 PMC6977799

[ref9] Gaugler J. James B. Johnson T. Reimer J. Solis M. Weuve J.. (2022). 2022 Alzheimer's disease facts and figures. Alzheimer's Dement. 18, 700–789. doi: 10.1002/alz.12638, PMID: 35289055

[ref10] Huang L. K. Chao S. P. Hu C. J. (2020). Clinical trials of new drugs for Alzheimer disease. J. Biomed. Sci. 27:18. doi: 10.1186/s12929-019-0609-7 31906949 PMC6943903

[ref11] Jiang C. Xu Y. (2014). The association between mild cognitive impairment and doing housework. Aging Ment. Health 18, 212–216. doi: 10.1080/13607863.2013.823376, PMID: 23919266

[ref12] Kartschmit N. Mikolajczyk R. Schubert T. Lacruz M. E. (2019). Measuring cognitive reserve (CR) - a systematic review of measurement properties of CR questionnaires for the adult population. PloS One 14:e0219851. doi: 10.1371/journal.pone.0219851, PMID: 31390344 PMC6685632

[ref13] León I. García-García J. Roldán-Tapia L. (2014). Estimating cognitive reserve in healthy adults using the cognitive reserve scale. PloS One 9:e102632. doi: 10.1371/journal.pone.0102632 25050711 PMC4106838

[ref14] Lista S. Molinuevo J. L. Cavedo E. Rami L. Amouyel P. Teipel S. J.. (2015). Evolving evidence for the value of neuroimaging methods and biological markers in subjects categorized with subjective cognitive decline. J. Alzheimer's Dis. 48 Suppl 1, S171–S191. doi: 10.3233/JAD-150202, PMID: 26402088

[ref15] Lu Y. Liu C. Yu D. Fawkes S. Ma J. Zhang M.. (2021). Prevalence of mild cognitive impairment in community-dwelling Chinese populations aged over 55 years: a meta-analysis and systematic review. BMC Geriatr. 21:10. doi: 10.1186/s12877-020-01948-3, PMID: 33407219 PMC7789349

[ref16] Mao G. Lu F. Fan X. Wu D. (2020). “China’s ageing population: the present situation and prospects” in Population change and impacts in Asia and the Pacific. New Frontiers in regional science: Asian perspectives. eds. Poot J. Roskruge M. (Singapore: Springer).

[ref17] McCleery J. Laverty J. Quinn T. J. (2021). Diagnostic test accuracy of telehealth assessment for dementia and mild cognitive impairment. Cochrane Database Syst. Rev. 2021:CD013786. doi: 10.1002/14651858.CD013786.pub2, PMID: 34282852 PMC8406800

[ref18] Overton M. Pihlsgård M. Elmståhl S. (2019). Prevalence and incidence of mild cognitive impairment across subtypes, age, and sex. Dement. Geriatr. Cogn. Disord. 47, 219–232. doi: 10.1159/000499763, PMID: 31311017

[ref19] Pichet Binette A. Vachon-Presseau É. Morris J. Bateman R. Benzinger T. Collins D. L.. (2021). Amyloid and tau pathology associations with personality traits, neuropsychiatric symptoms, and cognitive lifestyle in the preclinical phases of sporadic and autosomal dominant Alzheimer's disease. Biol. Psychiatry 89, 776–785. doi: 10.1016/j.biopsych.2020.01.023, PMID: 32228870 PMC7415608

[ref20] Qin J. He Z. Wu L. Wang W. Lin Q. Lin Y.. (2021). Prevalence of mild cognitive impairment in patients with hypertension: a systematic review and meta-analysis. Hypertens. Res. 44, 1251–1260. doi: 10.1038/s41440-021-00704-3 34285378

[ref21] Radler K. H. Zdrodowska M. A. Dowd H. Cersonsky T. E. K. Huey E. D. Cosentino S.. (2020). Rate of progression from mild cognitive impairment to dementia in an essential tremor cohort: a prospective, longitudinal study. Parkinsonism Relat. Disord. 74, 38–42. doi: 10.1016/j.parkreldis.2020.04.008, PMID: 32325394 PMC7587472

[ref22] Ren R. Qi J. Lin S. Liu X. Yin P. Wang Z.. (2022). The China Alzheimer report 2022. Gen. Psychiatr. 35:e100751. doi: 10.1136/gpsych-2022-100751, PMID: 35372787 PMC8919463

[ref23] Sha F. Zhao Z. Wei C. Li B. (2022). Modifiable factors associated with reversion from mild cognitive impairment to cognitively Normal status: a prospective cohort study. J. Alzheimer's Dis. 86, 1897–1906. doi: 10.3233/JAD-215677, PMID: 35253766

[ref24] Su N. Li W. Li X. Wang T. Zhu M. Liu Y.. (2017). The relationship between the lifestyle of the elderly in Shanghai communities and mild cognitive impairment. Shanghai Arch. Psychiatry 29, 352–357. doi: 10.11919/j.issn.1002-0829.217059, PMID: 29719346 PMC5925586

[ref25] Tarumi T. Rossetti H. Thomas B. P. Harris T. Tseng B. Y. Turner M.. (2019). Exercise training in amnestic mild cognitive impairment: a one-year randomized controlled trial. J. Alzheimer's Dis. 71, 421–433. doi: 10.3233/JAD-181175, PMID: 31403944

[ref26] Wang Z. Hou J. Shi Y. Tan Q. Peng L. Deng Z.. (2020). Influence of lifestyles on mild cognitive impairment: a decision tree model study. Clin. Interv. Aging 15, 2009–2017. doi: 10.2147/CIA.S265839 33149562 PMC7604452

[ref27] Wood H. (2016). Alzheimer disease: Meta-analysis finds high reversion rate from MCI to normal cognition. Nat. Rev. Neurol. 12:189. doi: 10.1038/nrneurol.2016.29, PMID: 26965671

[ref28] Xue J. Li J. Liang J. Chen S. (2018). The prevalence of mild cognitive impairment in China: a systematic review. Aging Dis. 9, 706–715. doi: 10.14336/AD.2017.0928, PMID: 30090658 PMC6065290

[ref29] Zhaoyang R. Sliwinski M. J. Martire L. M. Katz M. J. Scott S. B. (2021). Features of daily social interactions that discriminate between older adults with and without mild cognitive impairment. J. Gerontol. B Psychol. Sci. Soc. Sci. 79:gbab019. doi: 10.1093/geronb/gbab019 33528558 PMC10935459

