# Supplementary material for: A nomogram for individualized prediction of mild cognitive impairment in patients with subjective cognitive decline during physical examinations: a cross-sectional study
Source: Front Aging Neurosci. 2024 Jul 3;16:1443309. doi: 10.3389/fnagi.2024.1443309 (PMC11251993; doi:10.3389/fnagi.2024.1443309)
Supplement: Supplementary file 1 [file Table_1.DOCX]

***Supplementary Tables***

**Supplementary Table 1**

**Diagnostic Criteria: Expert Consensus on Preclinical SCD Diagnostic Procedures and Norms for AD in China (2020 Revision)**

**A. Diagnostic criteria for MCI: Neuropsychological criteria**

| MCI can be diagnosed if one of the following conditions is met: |
| --- |
| (1) Scores further than 1.0 SD from the mean (age-adjusted norm) on at least two neuropsychological tests in the same cognitive domain (memory, language, executive function). |
| (2) Scores on each cognitive domain (memory, language, executive function) of a neuropsychological test further than 1.0 SD from the mean (age-adjusted norm). |
| (3) An FAQ score of 9, reflecting impaired ability to perform at least three or more daily activities independently. |

**B. Diagnostic criteria for subjective cognitive decline in pre-MCI studies**

(1) Sustained decline in perceived cognitive function compared to the previous normal state, independent of acute events.

(2) After adjusting for age, sex, and years of education, exhibited normal performance on standard cognitive tests (in contrast to those with MCI and predementia).

Both (1) and (2) must be present.

Exclusion criteria:

(1) MCI, prodromal dementia AD or dementia

(2) Cognitive decline explained by psychiatric or neurological disorders * (except AD), medical conditions, drug side effects, or substance abuse

* Exclusion criteria do not include symptoms of anxiety or depression without meeting the diagnostic criteria for the corresponding disorder

**C. Characteristics that increase the likelihood of preclinical AD in SCD individuals:**

1. Subjective memory decline but no impairment in other cognitive functions

2. Onset time <5 years

3. Age of onset ≥60 years old

4. Concerns about cognitive decline

5. Self-perceived memory worse than that of people of the same age

Conditional studies further detect the following:

6. Cognitive decline confirmed by family or friends

7. Carrier of the apolipoprotein E ε4 allele (APOE ε4)

8. Evidence of an AD biomarker

**Abbreviations**

MCI: mild cognitive impairment; AD: Alzheimer's disease; AD: Alzheimer's disease; SCD: subjective cognitive decline.
